# Supplementary material for: Human-hippo conflicts around Lake Tana Biosphere Reserve, Ethiopia: Vulnerability of hippopotamus in human-dominated landscape
Source: PLoS One. 2023 Oct 5;18(10):e0291802. doi: 10.1371/journal.pone.0291802 (PMC10553267; doi:10.1371/journal.pone.0291802)
Supplement: S1 File — (DOCX) [file pone.0291802.s001.docx]

**Supporting information: Questionnaire**

Questionnaire: This is the questionnaire interview which was used to examine human-hippo conflicts around Lake Tana Biosphere Reserve, Ethiopia. The questionnaire interview was conducted with those local farmers who live near the vicinity of the lake. This information is highly confidential.

Thank you in advance!

1. Study site:____________ Village _________Date_______________

2.: Sex of respondent: M___ F ___

3. Age of respondent_________

4. Number of family member/household size of the respondent ____A. Male___ B. Female ___

4. What is your educational status? A. None B. Primary C. Secondary D. Religion

5. What is the source of your livelihood? A. Crop cultivation B. Livestock rearing C. Fishing D. Others (if any)___________

6. Could you enumerate the total number of domestic animals that you own?

A. Cattle_____ B. Sheep_____ C. Goat **_____** D Equine **____** E. Poultry_____ F. Others (if any)______

7. What types of crops do you grow on your farmland (starting from the most common cultivated to the least?) 1. ____________2. _____________ 3.___________4.___________5.__________6.__________

8. Could do mention the type of large mammals live in and around Lake Tana? __________________________________________________________________________________

9. Is there any human-hippos conflict around your village? A. Yes B. No. C. I don’t know

10. What are the causes of human-hippo conflict around your village? A. Crop damage B. Competition with livestock over grazing pastures C. Livestock injure/biting D. Aggression against humans F. Others (if any)______________________

11. What kind crops do hippos damage near your village? A. Maize B. Teff C. Finger millet D. Rice E. Others (if any)______________________

12. In which stage of crops do hippos damage most frequently? A. Seedling B. Vegetative C. Maturation

13. How is the extent of crop damage by hippos around your village? A. Severe B. Medium/less severe C. Insignificant

14. When do hippos damage your crops? A. At day hours B, At bight C. Both at day and night hours

15. How is the trend of crop damage by hippos year after year near around your village? A. Increasing B. Stable C. Decreasing . Why__________________________________________

16. What is the population size of hippos that inhabit Lake Tana around your village? ________________

17. How is the population trend of hippos near your village? A. Increasing B. Decreasing C. Stable D. I don’t know

18. What is your attitude towards hippos? A. Positive B. Negative C. Neutral.

19. What factors do you like or dislike hippos?________________________________________

20. Should hippos conserve in Lkae Tana? A. Yes B. No C. Neutral. Why________________

21. What are opportunity costs do you face due to human-hippo conflict? A. Sleeping loss B. Biting by mosquitoes/insects C. Infected with malaria D. Others (if any)__________________

22. Do local people kill hippos around your village? A. Yes B. No. C. I don’t know

23. Why do local people kill hippos? A. As revenge of crop damage or human/livestock injure B. For medicinal purpose C. Use of its skin for making ropes D. Others (if any)_______________

24. What kind of methods do you use to protect your crops from being damaged by hippos? Please mention them__________________________________________________________________

25. What kind of mitigation measures do you suggest to minimize the extent of human-hippo conflict around your village? ______________________________________________________

26. Do you have any other comments or suggestions that you like to add/make? ____________________________________________________________________________

Thank you again!!
