# Supplementary material for: Human-hippo conflicts around Lake Tana Biosphere Reserve, Ethiopia: Vulnerability of hippopotamus in human-dominated landscape
Source: PLoS One. 2023 Oct 5;18(10):e0291802. doi: 10.1371/journal.pone.0291802 (PMC10553267; doi:10.1371/journal.pone.0291802)
Supplement: Attachment — Submitted filename: PlosOneReview22May2023.pdf [file pone.0291802.s003.pdf]

This article is on human-hippo conflict in an understudied region of Ethiopia. The authors did a preliminary hippo survey to count the hippos near different villages and then used questionnaires to understand how the locals in each of the villages respond to human-hippo conflict.

The authors found that there is significant human-hippo conflict and that most locals would prefer the hippo were not there. They found that hippos were responsible for significant crop damage. The authors recommend a proper land use zonation system around the LTBR, more awareness programs for the locals and potentially alternative crop production schemes.

I enjoyed reading the manuscript. I have a few suggestions that I hope will help improve it.

Line 12 – change “impact” to “impacts”.

Line 28 – unclear how the population size was 243.5? Maybe drop a significant digit since you can’t really have half a hippo.

Line 39-40 – Maybe change the beginning of the sentence to, “Conflict often occurs when human settlements….”.

Line 41-43 – Maybe restructure the sentence in a little bit. “People have many interests to utilize the resources associated with wildlife occupied wetland habitats which complicates conservation issues.”

Line 48 – change “utilizing” to “utilization”.

Line 52 – Suggest restructuring sentence like this…”One of the wetland mammals of Ethiopia who frequently spends time in and at the edge of water bodies is the hippopotamus (Hippopotamus amphibious).”

Line 58 – add a citation at the end of this sentence.

Line 59 – change “Megaherbivores” to “Megaherbivore”

Line 61 – add a citation for the sentence about human-wildlife conflict with elephants and hippos.

Line 66 – change “few studies were conducted” to “few studies have been conducted”

Line 66-68 – at the end of the sentence, add a “but see” and cite this paper - https://link.springer.com/article/10.1007/s43621-022-00077-8. This is so that you’re acknowledging that it is poorly documented, but here is one paper that does document it.

Line 71 – can you add a citation for this…that the wetland provides lots of goods and services?

Line 79 – make sure you put the ethical statement in Plos One box that says, “Ethics Statement”…on page 3 of the PDF. You currently have it listed as N/A, which worried me. Since you would need board review for human surveys.

Line 137 – change “become” to “come”

Line 186 – I would remove the “half a hippo” and just keep the number at 243. Having that extra significant digit isn’t really necessary and may just confuse people. Or you could round up to 244.

Table 3 – I'm confused about the "Mean estimate by respondents". I think I understand it, but it might be helpful to add a column with the number of respondents from each of the villages, so that the reader will have an idea of how accurate it is..or you could add a standard deviation too for each of the villages.

Line 218 – change "for their safe habitats" to "for their safety."

Line 220 – change "occupied" to "occupies".

Line 226 – change "Wetland is" to "Wetlands are"

Line 227 – change "overgrazing" to "overgrazing by livestock"

Line 231 – change "studies" to "sociological studies".

Line 242 – change [23] to actually list the authors, "Kanga et al" and add the [23] at the end of the sentence.

Line 244 – change "proximate" to "proximity" and change "with" to "near" and then in the next line, change "neat" to "near".

Line 256 – alter the sentence a bit so it is more clear. How about this, "Similarly, damage by megaherbivore mammals like elephants lead to a negative perception of the species among local people across Africa [10,25,26]." And then make the rest a new sentence like this, "Drop damage by elephants hurt the local social economy and lead to reduced local tolerance for species around Moukalaba-Doudou National Park in Gabon [9]".

Line 264 – change "their broad feet" to "trampling".

Line 267 – I think the citation you mean for this sentence is [9] and not [10]? Check it please.

Line 284 – change "conservations" to "conservation".

Line 288 – delete "problems".

Line 300 – change "permission" to "permitting".

I would also suggest that you upload the data to a free archive so that you do not have to rely on communicating with anyone to provide the data.

I also suggest that you upload the survey questions as supplementary material.
