# Supplementary material for: Human-hippo conflicts around Lake Tana Biosphere Reserve, Ethiopia: Vulnerability of hippopotamus in human-dominated landscape
Source: PLoS One. 2023 Oct 5;18(10):e0291802. doi: 10.1371/journal.pone.0291802 (PMC10553267; doi:10.1371/journal.pone.0291802)
Supplement: Attachment — Submitted filename: Human-Hippo Conflict.docx [file pone.0291802.s002.docx]

Comments

This is an interesting and well written paper. The abstract, methods and discussion have been presented clearly. I recommend it for publication as it offers a significant case study in the area of human-wildlife conflict.

However, there are a few issues the authors might consider looking at. Not taking away what has been done, but just to help add more information and strengthen the paper.

Abstract

A well written abstract which outlines the key argument, research methodology and research findings. I would suggest that the findings be summarised into one or two statements and leave the rest especially percentages to the main discussion.

For instance lines 23-27 –could be captured as - Most respondents (range 90 to 93%) complained about high crop damage suggesting that hippos be eliminated from the region.

**Introduction**

Line 39-40 – It is often results in when …. It is not sounding well

First paragraph – there is mention of conservation but what this conversation is about or means is not explained

From lines 1-51 – cause of human-wildlife conflict is given to human population and their activities – is the Ethiopian government not involved in causing conflict e.g. it controls how land is parcelled or used by its people – is the government responding to the demands of an increased population to assist with reducing conflict? The issue is - doesn’t the government have a role in this – directly or indirectly – can be mentioned in passing then place emphasis on the focus of the study

The introduction is properly contextualised – it highlights the dynamics and changes relating to human-wildlife conflict in Africa in general and Ethiopia in particular.

**Materials and Methods**

The study area is well described – key points delineating the study area are given which will help the reader to follow arguments and findings that will be presented later.

Line 121 – it would be beneficial to mention the number of households and villages involved

Line 124 – what informed the use of chance encounter as a tool of collecting data and not others? How did it benefit the study?

Line 136-37 - … when hippos attempt to become out … attend to this part

Line 145-46 – is there any particular reason why female respondents were 3.9% yet agriculture is mainly practiced by women including fetching water where conflicts tend to occupy?

155-56 – no respondents claimed human provocation as a cause of hippo aggression – if not, why so? Or the authors did not ask this question? As it stands humans presented themselves as the victims?

Line 172-73 – if hippo crop damage happened mostly at night – yet the authors did not count the hippo population at night when the majority would be out – the question is - did that not affect the figures they got by conducting survey of hippos in the early morning and late afternoon – by not observing hippos during the night, the authors did not get a full picture of the conflict – did this not affect the recommendations of the paper?

192 – Table – the authors do not state methods used by respondents to count hippos to get their number? – why did the authors not compare their gathered statistics with that of managers of the area, i.e., wildlife management authorities

201-202 – is there a difference between local farmers and residents of the area? – if so, what informs their different perceptions about hippos?

202-05 – they witnessed 2 dead hippos yet arrived at the conclusion that there is a high intense degree of human-hippo conflict – how did you arrive at this position after seeing only 2 dead hippos? Did you ascertain if they died of human-hippo conflict or natural cause or hippo-hippo conflict?

**Discussion**

239 – cultivation in wetlands, river banks – more could be said about its impact to the environment and conservation of the same – when we talk of conservation – what is it to be conserved? Hippos? Agricultural activities? Wetlands? Rivers from silting? Soil erosion?

A general observation is that in the discussion nothing is mentioned about managers of the wetland or wildlife – did the respondents not include the role of managers in the discussion or the authors deliberately left them out of discussion – I believe local residents’ response to human-hippo conflict is also informed by the authorities’ agency or lack of it in the matter – what is the situation in this case?

291 – creating alternative job opportunities – who should do this? It is not stated – is this coming to the government? If so, is the government of Ethiopia able to perform this task considering the rate of unemployment in the country? It means opening new industries or companies and is this sustainable? The authors are best suited to answer this, but if answers to these questions are negative, what does it mean to the recommendation?
