# Supplementary material for: Human-hippo conflicts around Lake Tana Biosphere Reserve, Ethiopia: Vulnerability of hippopotamus in human-dominated landscape
Source: PLoS One. 2023 Oct 5;18(10):e0291802. doi: 10.1371/journal.pone.0291802 (PMC10553267; doi:10.1371/journal.pone.0291802)
Supplement: Attachment — Submitted filename: Zewdu Kifle_response to the reviewers.docx [file pone.0291802.s004.docx]

July 10 2023

Ethiopia

Ref.: PONE-D-23-07127

Dear Frank O. Masese, Ph.D,

Academic Editor, PLOS ONE

This is a revision of our manuscript entitled “Human-Hippo Conflicts around Lake Tana Biosphere Reserve, Ethiopia: Vulnerability of Hippopotamus in Human-Dominated Landscape "by Zewdu Kifle, Workiyie Worie Assefa, and Amera Moges. On 13 Jun 2023, I received revision request in order to reconsider for publication. You invited me to submit a revised version of the manuscript that addresses the points raised during the review process. Hence, I addressed all the points and comments raised by the reviewers’ within this revised version of the manuscript.

Below there are lists of the revisions.

Sincerely,

Zewdu Kifle, Ph.D,

Correspondence Author

**Response for Editor and reviewers**

Thank you for your critical comments. I highlighted revisions with green color. In addition, I highlighted with red color and strikethrough for those deleted letters, words, phrases or sentences.

Thank you!

**Response to the Editor**

1. Okay! We think that our manuscript meets PLOS ONE's style requirements.
2. Well! All relevant data are available within the paper.
3. Thank you for your comment on Figure 1. We presented our prepared map in this resubmitted manuscript.
4. Okay! We reviewed our reference list. It is complete and correct.

**Response for reviewers**

Reviewer #1

General comments: Thank you for appreciating the manuscript and its importance to understand human-hippo conflict in the region. We corrected those ideas based on your interesting comments.

Specific comments:

1. Line 12: Okay! W changed it.
2. Line 28: Okay! We recommended to do further survey to determine accurate population size of hippos in the region.
3. Line 39-40: Well! Thank you!
4. Line 41-43: Thank you! We restructured the sentence.
5. Line 48: We changed it.
6. Line 52: Well! We restructured the sentence.
7. Line 58: Well! We added the citation.
8. Line 59: Okay! Thank you! We changed it.
9. Line 61: We added the citation here.
10. Line 66: Thanks! We changed it.
11. Line 66-68: Okay! We added this paper.
12. Line 71: Okay! We added a citation here.
13. Line 79: Okay! Thank you!
14. Line 137: Well! We changed it.
15. Line 186: We removed the digit.
16. Table 3: Thank you! We added number of respondents and Standard deviation (SD) for each village.
17. Line 218: Okay, We changed it.
18. Line 220: Okay, We changed it.
19. Line 226: Well, we changed it.
20. Line 227: Okay, We added it.
21. Line 231: Okay, We changed it.
22. Line 242: Okay! We corrected it.
23. Line 244: Thank you! We corrected this sentence.
24. Line 256: Okay! We did it.
25. Line 264: We changed it.
26. Line 267: Okay, Thanks!
27. Line 284: We changed this word.
28. Line 288: Well! We deleted it.
29. Line 300: We changed it.

Reviewer #2

General comments: Thank you for admiring the manuscript and its offers in the area of human-wildlife conflict. We added more information based on your interesting comments to strengthen the paper.

Specific comments:

Abstract:

1. Okay, W summarized the findings based on your comment.

Introduction:

1. Line 39-40: Okay! We corrected it.
2. Lines 1-51: We wrote this part in more detailed.

Materials and Methods:

1. Okay! We pointed key points on the map.
2. Line 121: Okay! We mentioned villages involved in data collection section, and the numbers of respondents here.
3. Line 124: Chance encounter avoids biases. We cited about it in the text. This method is very important to collect data from randomly encountered respondents within a short period of time and it is also unbiased tool.
4. Line 136-37: Okay! We corrected this part. We changed ‘become out’ to ‘come out’.
5. Line 145-46: We have no particular reason. But in Ethiopian culture females give priorities for their husband. Thus, when we got both, we interviewed the males than the females. In addition, we encountered males in the field nearby the lakeshore in most cases too. Thus, we had no any particular reason why female respondents were 3.9% other than this.
6. Line 155-56: We didn’t ask about human provocation as a cause of hippo aggression.
7. Line 172-73: Okay! Thank you, you right, the majority of hippos would be out at night. In this paper, we recommend to conduct hippo population survey at night and to get a full picture of the conflict in the region. Thus, such recommendation is very helpful for our paper by providing future population survey and human-hippo conflict observation directions. Thus, our survey doesn’t affect the paper.
8. Line 192 –Table: During our interview questionnaire surveys, we asked the respondents to estimate number of hippo populations inhabiting around their villages. Since, local people frequently are encountered with hippos around their farmlands; they are good sources to estimate hippos around their villages. We asked managers (wildlife management authorities) of the number of hippos in the LTBR. But, they didn’t have data about the population size (number) of hippos in the area. Thus, we couldn’t compare our gathered hippo statistics with the data of managers of the area. Our survey is the first in the area.
9. Line 201-202: There is no a difference between local farmers and residents of the area. But to avoid confusion, we deleted residents and replaced by the local farmers.
10. Line 202-205: you see, we observed two freshly dead hippos in our single survey. If we follow such killings in year round or more, we can get more dead hippos. We ascertained the killings by asking the local people. They informed us that the local farmers killed them through automatic gun firing.

Discussion:

1. Line 239: All natural resources should be conserved. Thus, such cultivation trends towards wetlands and river banks could have a great impact to the environment and wildlife conservation.

The managers of LTBR know the problem (human-hippos conflict) in the area. But, they do not have any scientific data to incorporate in this paper or discussion parts.

2. Line 291: The government should create alternative job opportunities to minimize human demand and load on the hippo habitats.
